# Preclinical Deposition of Pathological Prion Protein in Muscle of Experimentally Infected Primates

**DOI:** 10.1371/journal.pone.0013906

**Published:** 2010-11-11

**Authors:** Susanne Krasemann, Melanie Neumann, Markus Geissen, Walter Bodemer, Franz-Josef Kaup, Walter Schulz-Schaeffer, Nathalie Morel, Adriano Aguzzi, Markus Glatzel

**Affiliations:** 1 Institute of Neuropathology, University Medical Center Hamburg-Eppendorf, Hamburg, Germany; 2 German Primate Center, Göttingen, Germany; 3 Institute of Neuropathology, University Hospital Göttingen, Göttingen, Germany; 4 CEA, IBitec-S, Service de Pharmacologie et dlmmunoanalyse, CEA/Saclay, Gif sur Yvette, France; 5 Institute of Neuropathology, University Hospital Zurich, Zurich, Switzerland; Uppsala University, Sweden

## Abstract

Prion diseases are transmissible fatal neurodegenerative disorders affecting humans and animals. A central step in disease progression is the accumulation of a misfolded form (PrP^Sc^) of the host encoded prion protein (PrP^C^) in neuronal and non-neuronal tissues. The involvement of peripheral tissues in preclinical states increases the risk of accidental transmission. On the other hand, detection of PrP^Sc^ in non-neuronal easy-accessible compartments such as muscle may offer a novel diagnostic tool. Primate models have proven invaluable to investigate prion diseases. We have studied the deposition of PrP^Sc^ in muscle and central nervous system of rhesus monkeys challenged with sporadic Creutzfeldt-Jakob disease (sCJD), variant CJD (vCJD) and bovine spongiform encephalopathy (BSE) in preclinical and clinical stage using biochemical and morphological methods. Here, we show the preclinical presence of PrP^Sc^ in muscle and central nervous system of rhesus monkeys experimentally infected with vCJD.

## Introduction

Prion diseases are transmissible neurodegenerative disorders characterized by neuronal loss, astrocytosis and deposition of the pathogenic isoform (PrP^Sc^) of the cellular prion protein (PrP^C^). Conversion of physiological PrP^C^ into pathogenic PrP^Sc^ plays a major role in disease pathophysiology and PrP^Sc^ is a principal component of prion infectivity [1], [2], [3]. PrP^Sc^ differs from PrP^C^ in its increased ß-sheet content, which renders it relatively resistant to proteolytic digestion [4], [5].

Prion diseases affect humans and animals alike and both human-to-human and animal-to-human transmission may occur [6]. Human prion diseases include sporadic, genetic and acquired forms [7], [8]. Different prion-isolates show specific clinical and biochemical traits which are referred to as prion strains [9]. Sporadic Creutzfeldt-Jakob disease (sCJD) has unknown aetiology and may result from spontaneous conversion of PrP^C^ to PrP^Sc^ [10]. Genetic Creutzfeldt-Jakob disease (gCJD) co-segregates with mutations in the gene encoding the prion protein and is inherited autosomal dominantly [11], whereas acquired forms are caused by exposure to infectious human prions during medical or neurosurgical procedures (iatrogenic Creutzfeldt-Jakob disease, iCJD) or a non-human prion source such as bovine spongiform encephalopathy (BSE)-prions (variant Creutzfeldt-Jakob disease, vCJD) [12]. Interhuman transmission of vCJD through blood transfusions has occurred in several instances [6], [13]. In contrast, no such transmissions have been reported with other human prion diseases such as sCJD. This demonstrates that peripheral tissues of vCJD infected individuals harbouring relatively low prion titer may lead to prion transmission following host-adaption.

Non-human primate studies using macaques as model organism have been valuable in elucidating pathophysiology of prion diseases [14], [15], [16], [17]. Primate studies routinely assess animals in clinical disease states, thus distribution of PrP^Sc^ during subclinical disease has not been investigated in primates [16]. Detailed knowledge on the distribution of PrP^Sc^ in model organisms closely resembling the human situation is important because it allows evidence based decisions aimed at limiting the spread of vCJD through iatrogenic procedures [18], [19], [20]. Furthermore, knowledge on the temporal kinetics of PrP^Sc^ accumulation in tissue compartments, which are easily accessible such as muscle may help in devising novel approaches for diagnostic tests, aimed at detecting prion diseases at subclinical stage.

Muscle biopsy has been proposed as a novel tool for diagnosing human prion diseases [21]. PrP^Sc^ is detectable in muscle of a wide range of human prion diseases and data from rodent studies show presence of muscular PrP^Sc^ in preclinical stages [22], [23], [24], [25].

We therefore systematically investigated the deposition of PrP^Sc^ in muscle and central nervous system in rhesus monkeys challenged with sCJD, vCJD and BSE-prions in preclinical and clinically diseased animals using ultrasensitive western blotting, ELISA and PET-blot. Our results show subclinical deposition of PrP^Sc^ in muscle of vCJD infected primates. These results should be taken into consideration when devising strategies aimed at limiting iatrogenic transmission of vCJD. The variability of detection of PrP^Sc^ in muscle of clinical and subclinical primates suggests inhomogeneous distribution and challenges the idea of employing muscle biopsy as a routine diagnostic tool in prion diseases.

## Results

### Clinical prion disease following intraperitoneal application of vCJD and BSE

To investigate if intraperitoneal prion inoculation leads to the development of clinical prion disease, we infected rhesus monkeys with vCJD, sCJD and BSE-prions or saline intraperitoneally. Animals were monitored until signs of prion disease were present and the experiment was terminated 335 weeks post inoculation (wpi). Incubation times until clinical signs of prion disease were 172 wpi for the vCJD infected animal and 212 wpi for the BSE infected primate, whereas the sCJD infected primate did not show any signs of prion disease until the experiment was terminated (table 1).

**Table 1:** Primates included in the study.

Group	wpi	Clinical disease	Muscular PrP^Sc^						CNS PrP^Sc^		
			Tongue		Arm		Heart				
			ELISA	WB	ELISA	WB	ELISA	WB	ELISA	WB	PET
vCJD	132	no	−	−	−	−	−	−	+	+	+
	144	no	+	+	−	−	+	+	+	+	+
	158	no	+	+	+	+	−	−	+	+	+
	172	yes	+	+	−	+	+	−	+	+	+
sCJD	243	no	−	−	−	−	−	−	−	−	−
	335	no	n.d.	−	n.d.	−	n.d.	−	n.d.	−	−
BSE	107	no	−	−	−	−	−	−	−	−	−
	212	yes	+	+	−	−	−	−	n.d.	+	+
Mock	106	no	n.d.	−	n.d.	−	n.d.	−	n.d.	−	−
	222	no	−	−	−	−	−	−	n.d.	−	−

### Exponential increase of PrP^Sc^ in brains of vCJD infected primates

To investigate pattern and amounts of PrP^Sc^ deposition in preclinical primates, we performed a time course study in rhesus monkeys infected with vCJD, sCJD and BSE or saline intraperitoneally. For vCJD, animals were sacrificed at three preclinical time points (132 wpi (n = 1), 144 wpi (n = 1), 158 wpi (n = 1)), and one clinical time point (172 wpi (n = 1)). For sCJD one monkey was taken at 243 wpi. The other sCJD challenged monkey was sacrificed at 335 wpi and both failed to develop clinical prion disease within the observation period. For BSE, one animal was taken preclinically (107 wpi) and one animal clinically (212 wpi) (table 1). In order to assess PrP^Sc^ deposition in the central nervous system (CNS) we performed standard and ultrasensitive NaPTA-enhanced Western blot analysis of cerebellum and frontal cortex.

**Figure 1:**
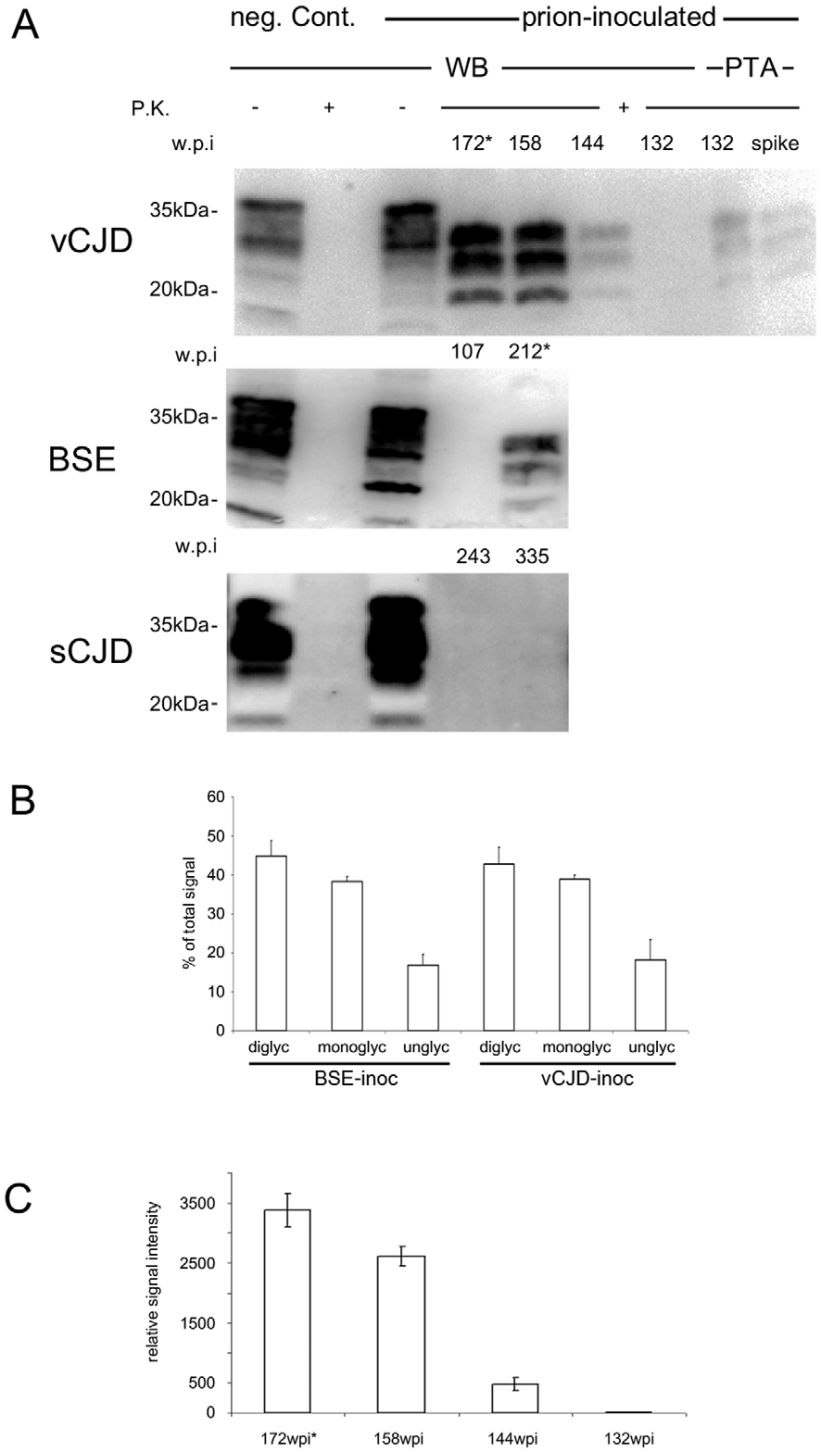
Biochemical analysis of PrP^Sc^ in the CNS. A) Western Blot analysis for PrP^Sc^ (frontal cortex) of vCJD, BSE and sCJD infected animals. PrP^Sc^ could be demonstrated in the brains of preclinical vCJD and prion-diseased vCJD and BSE inoculated animals. In the vCJD cohort, PrP^Sc^ is detectable (using NaPTA) in subclinical state 40 weeks before onset of symptoms. sCJD inoculated macaques did not show PrP^Sc^ at any time point in cerebellum (data not shown) and frontal cortex in conventional as well as NaPTA enhanced Western blot. (* indicates prion-diseased animals). B) PrP^Sc^-glycotype analysis demonstrates comparable glycotypes of vCJD and BSE when transmitted to primates. Densitometric measurement of relative band intensities for di-, mono- and unglycosylated form of PrP^Sc^ is shown in % of total signal. C) Quantification of PrP^Sc^-signal shows initial exponential increase of PrP^Sc^ until 158 wpi when PrP^Sc^ levels off. Relative amounts of PrP^Sc^ are shown in arbitrary units as quantified in three independent experiments.

PrP^Sc^ could be detected in all preclinical and clinical vCJD infected macaques. In contrast, PrP^Sc^ could only be detected in the clinical BSE diseased animal, whereas no PrP^Sc^ deposition was detectable in a preclinical BSE monkey as well as in both preclinical sCJD infected animals (figure 1A, B).

The setup of the study allowed us to assess temporal development of PrP^Sc^ accumulation in the CNS of vCJD-infected monkeys. PrP^Sc^ could be detected as early as 132 wpi in vCJD inoculated primates and the amount of PrP^Sc^ increased until clinical disease (figure 1A). Densitometric analysis of western blots showed exponential increase of PrP^Sc^ until 158 wpi, whereas PrP^Sc^ amounts only increased moderately from 158 to 172 wpi (figure 1C). Strain properties of BSE and vCJD as reflected by the glycoprofile and running behaviour of the unglycosylated core fragment of PrP^Sc^ remained unchanged (figure 1A, B). vCJD and BSE-inoculated primates showed comparable sizes of unglycosylated PrP^Sc^ core fragments and compatible glycosylation profiles indicating congruent PrP^Sc^-types (figure 1A, B).

**Figure 2:**
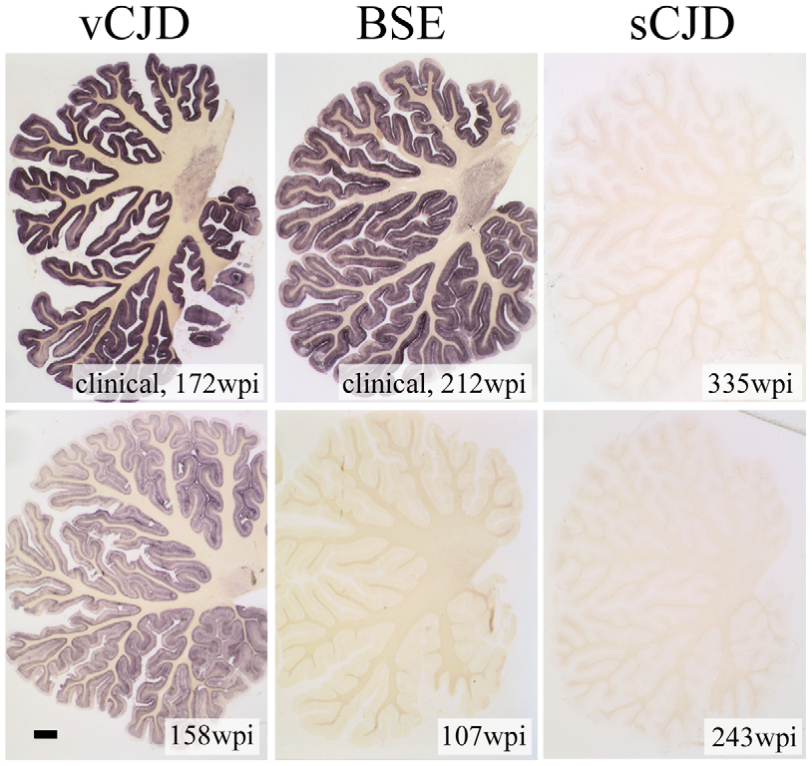
PET blots of cerebellum. PET blots show prominent deposition of PrP^Sc^ in granular and molecular cell layers in clinically diseased vCJD and BSE inoculated animals whereas the subclinical vCJD inoculated primate only shows faint PrP^Sc^ in granular and molecular cell layers (scale bar in lower left image 1 mm).

Western blot findings could be confirmed by PET-blot of cerebellum showing abundant deposits of PrP^Sc^ in monkeys challenged with vCJD and BSE-prions at terminal prion disease and faint PrP^Sc^ deposits in a subclinical, vCJD challenged animal (figure 2). No PrP^Sc^ could be detected in cerebellum in either of the sCJD-infected monkeys as well as the preclinical BSE-challenged primate.

### Preclinical detection of PrP^Sc^ in muscle of vCJD inoculated primates

**Figure 3:**
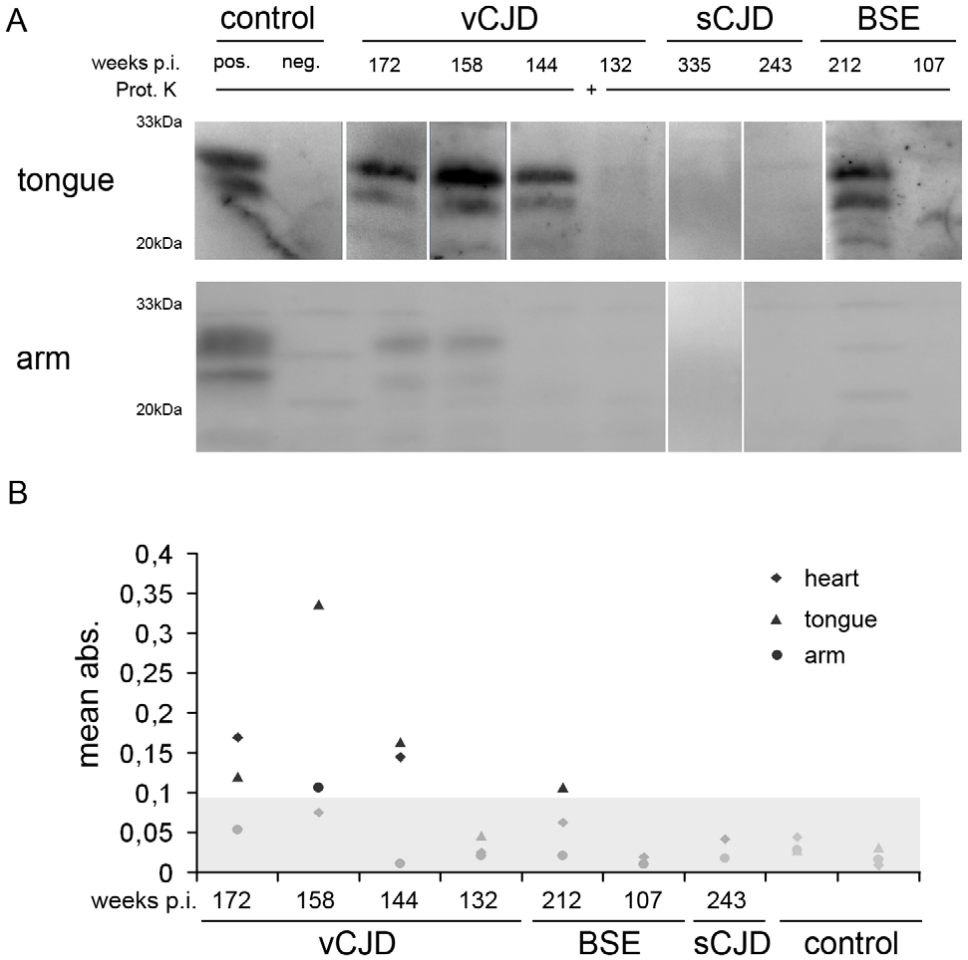
Preclinical detection of PrP^Sc^ in muscle of vCJD inoculated primates. A) Western Blot analysis of PrP^Sc^ from NaPTA precipitated arm and tongue tissue of sCJD, vCJD and BSE infected primates and controls show PrP^Sc^ in arm and tongue of subclinical vCJD challenged monkeys and in clinically affected vCJD and BSE inoculated animals. sCJD infected macaques did not show any detectable PrP^Sc^ in arm or tongue. B) Detection of PrP^Sc^ in arm, tongue and heart tissue from sCJD, vCJD and BSE infected primates and controls by ELISA. The scatter graph shows values (mean of two independent experiments) for arm (circle), tongue (triangle) and heart (diamond). The cut-off is indicated by grey shade and values below were considered as negative. PrP^Sc^ could be detected in tongue, arm and heart of healthy and clinically diseased vCJD inoculated animals. In BSE infected animals PrP^Sc^ could only be detected in tongue tissue and in sCJD infected animals no PrP^Sc^ could be detected.

To assess whether and to what extent PrP^Sc^ can be found in muscle we determined PrP^Sc^ contents using NaPTA-enhanced Western blot analysis and PrP^Sc^ specific ELISA (summarized in table 1). At 132 wpi, the earliest time point assessed for vCJD, no PrP^Sc^ could be detected in either western blot or ELISA of arm, tongue and heart (figure 3, western blot data for heart not shown). In contrast, at 144 wpi, PrP^Sc^ appeared in heart and tongue muscle but not in arm muscle with ELISA and western blot (figure 3, western blot data for heart not shown). At 158 wpi, PrP^Sc^ was demonstrated in arm and tongue by ELISA and western blot (figure 3A, B), whereas PrP^Sc^ was below detection limit by either method in heart (figure 3A, B). In the clinical diseased animal from the vCJD cohort, peripheral PrP^Sc^ deposits were abundant in tongue tissue when measured by western blot and ELISA, yet PrP^Sc^ in heart could only be detected by ELISA and PrP^Sc^ in arm tissue only by western blot. In the BSE cohort, no PrP^Sc^ could be found preclinically in arm, tongue or heart muscle using either method. The clinically affected BSE challenged primate showed unambiguous deposition of PrP^Sc^ in tongue tissue only (figure 3A, B). No PrP^Sc^ was observable in arm or heart. Both sCJD infected primates did not show any PrP^Sc^ in the investigated muscle tissue when assessed by western blot or ELISA (figure 3A, B, table 1).

### PrP^Sc^ deposits are associated to nerve fibers as well as to myocytes in tongue tissue

**Figure 4:**
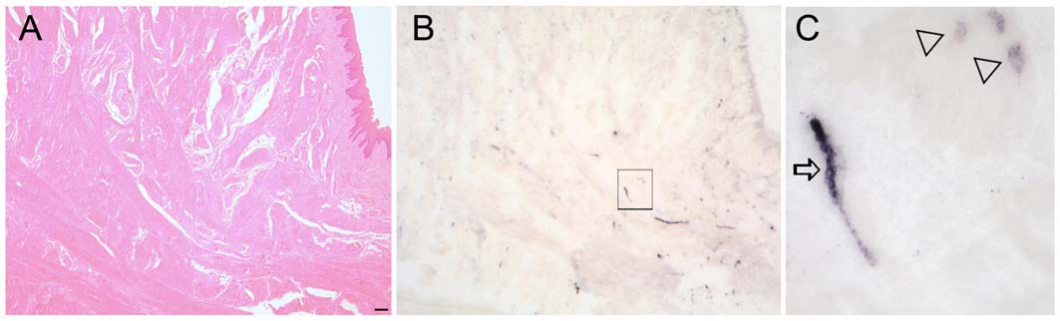
Morphological analysis of tongue reveals PrP^Sc^ deposition in nerve fibers and myocytes. A) Histological analysis of tongue from the clinical, vCJD infected macaque shows muscle fibers intermingled with peripheral nerves (Haematoxilin and Eosin staining, scale bar 50 µm). B, C) PET blot (consecutive section from A) showing labelling of PrP^Sc^. Higher magnification (C) of the insert shown in (B) revealed PrP^Sc^ staining of longitudinal nerve fibers (arrow) and adjacent myocytes (triangles).

To investigate the distribution of PrP^Sc^ in tongue tissue of vCJD infected primates we used PET blotting. Of the four investigated animals only one showed detectable PrP^Sc^. Tongue tissue from this clinically diseased animal (172 wpi) was shown to contain PrP^Sc^ by western blotting and ELISA (figure 3A, B). PrP^Sc^ labelling occurred in linear fashion in addition to dot like deposits. By directly correlating PET blot data with information from consecutive sections processed for histology we were able to identify an association of PrP^Sc^ with nerve fibers (linear deposits, figure 4B, C) and myocytes (dot like deposits, figure 4B, C).

## Discussion

The occurrence of human-to-human transmission of vCJD prions trough medical interventions has lead to concerns regarding the further development of the vCJD epidemic [26], [27]. Although a wealth of studies have provided us with detailed information on the temporal and spatial kinetics of prion diseases in rodents, there is an ongoing controversy on the preclinical distribution of prion infectivity and PrP^Sc^ in human prion diseases [28], [29]. Furthermore, the detailed analysis of PrP^Sc^ distribution in preclinical prion disease in an animal model closely resembling the human situation is a prerequisite for the validation of novel diagnostic tools such as muscle biopsy [16].

Non-human primate models have been used to determine transmission efficiency of sCJD, iCJD, vCJD or BSE [14], [15], [16]. Infectious brain homogenates have been applied *intracerebrally*, *intravenously*, *orally* or *intratonsillary*. The transmissibility of human prion diseases to non-human primates could be proven in any case [14], [15]. With our study we show that the intraperitoneal route, which is widely used in rodent models of prion diseases, is also effective in transmitting prion diseases in primates. Incubation time to clinical prion disease after intraperitoneal inoculation of BSE was comparable with oral infection [16], [30] with 48 months versus 47, 51 and 63 months respectively. For vCJD, no data are available for oral prion application. Incubation times were 25 to 37 month after intratonsillar and/or intracerebral inoculation [30]. In our study, one animal developed clinical disease after 40 months. Three other animals were taken preclinically 30, 32 and 34 months after prion challenge and PrP^Sc^ deposition could be readily shown in brain tissue of all four monkeys. PrP^Sc^ could not be detected in any of the investigated tissues using various methods in two primates intraperitoneally inoculated with sCJD, 56 and 77 month post inoculation. In contrast, Herzog [30] could show efficient transmission of sCJD with clinical prion disease 56 month post infection. Besides disparity in the administration of prions, these differences may be attributed to variations in prion titers of the inocula.

The shortening of incubation time in our experiment between BSE and vCJD inoculated macaques could be due to vCJD-strain adaptation or differences of titers of infectious prions present in inocula. Since prion titers were not determined in samples used in this study, we cannot rule out that these differences are due to prion titer variations. Nonetheless, other studies attribute shorting of incubation times between BSE and vCJD to strain adaptation effects using either intravenous or intracerebral routes of inoculation [14].

However, none of the former studies addressed the question of preclinical PrP^Sc^ distribution and the temporal development of PrP^Sc^ deposition. In our study we could show a drastic increase of PrP^Sc^ in the CNS of vCJD inoculated macaques over time. Interestingly, PrP^Sc^ accumulation seems to saturate the end of the incubation time. This fact is also known from rodent models of prion infection [31], but had never been demonstrated in primates before.

The peripheral distribution of PrP^Sc^ has been well characterized in rodent models of prion diseases [32], [33]. Deposition of PrP^Sc^ in muscle tissue had been shown in terminally sick hamsters experimentally infected with scrapie [25], mice infected with mouse adapted BSE and vCJD [24] as well as in sheep with naturally occurring scrapie [34], [35]. Preclinical PrP^Sc^ deposition in muscle tissue could be demonstrated in experimentally infected hamsters [36]. No preclinical PrP^Sc^ could be detected in heart muscle [36]. PrP^Sc^ had also been detected in muscle of patients suffering from sCJD, vCJD and iCJD [20], [23]. Moreover, the tissue distribution of PrP^Sc^ had been studied intensively in clinically diseased vCJD patients where PrP^Sc^ could be detected in limb, but not in tongue muscle [37], [38]

Here we show the deposition of PrP^Sc^ in muscle tissue of subclinical vCJD challenged primates. In agreement with other studies, PrP^Sc^ deposits within the musculoskeletal system showed patterns that are consistent with nerve fibers, yet we could also observe PrP^Sc^ in structures resembling myocytes [23]. The fact that highly innervated tongue muscle harbours more PrP^Sc^ than arm muscle points to prominent involvement of nerve fibers in the tongue not only in prion neuroinvasion [39] but also in PrP^Sc^ accumulation. Quantification of PrP^Sc^ in muscle using Western blotting or ELISA is variable. This may be due to inhomogeneous distribution of PrP^Sc^ in the muscular compartment as visualized by *in situ* detection methods for PrP^Sc^ such as PET blotting.

In summary, we could show that (i) PrP^Sc^ builds up exponentially in the CNS of vCJD infected primates, (ii) PrP^Sc^ is detectable in both CNS and the muscular compartment preclinically in vCJD challenged rhesus monkeys, (iii) PrP^Sc^ distribution in muscle tissue of clinical and subclinically infected primates is inhomogeneous.

These data should be taken into consideration when devising appropriate measures against iatrogenic transmission of prion diseases or when employing muscle biopsy in diagnosing human prion disease [15], [21], [23], [26]

## Materials and Methods

### Animals

All procedures involving animals were performed in accordance with European and German legal and ethical regulations (which are in line with the recommended practices of the use of non-human primates in research) and approved by the responsible boards and authorities. Captive-bred rhesus macaques (Macacca mulatta) were checked for absence of common primate pathogens and were inoculated intraperitoneally with 10 ml of a 10% homogenate in PBS of either BSE (mixture of cerebral cortex specimen from five clinically BSE affected cattle) sCJD (prion protein gene codon 129 MM, PrP^Sc^ type 2 according to [40]) and vCJD (prion protein gene codon 129 MM, PrP^Sc^ type 4 according to [40]; kindly provided by John Collinge, London, both cerebral cortex). Control animals received saline only. Animals were sacrificed at defined time points post inoculation and a subgroup of animals were allowed to progress to clinical prion disease (see table 1). These animals were sacrificed 4 weeks after onset of first clinical symptoms of prion disease (slowness, weight loss, trembling). The study was terminated 335 weeks post inoculation.

Tissues were either fixed in 4% formalin and prepared for histological examination or snap-frozen in liquid nitrogen and stored at −80°C for biochemical analyses.

### Western Blot analysis

For Western blot analysis, brain samples (Cerebellum, frontal cortex) were homogenised (FastPrep FP120, Qbiogene, Cedex, France) at 10% (weight/volume, w/v) in buffer (150 mM NaCl, 1 % NP-40, 0.5 % DOC, 0.1 % SDS, 50 mM Tris-HCl pH 8.0) and 0.1 mg wet brain was digested with proteinase K (PK) (20 µg/ml) for 45 min at 37°C. Digestion was stopped by adding 10× sample buffer and boiling for 10 min. Samples were loaded on a 12% SDS-PAGE gel transferred to PVDF membranes, blocked for 1 hour at room temperature in protein-free blocking buffer (Thermo Scientific, Rockford, USA) and incubated overnight at 4°C with monoclonal anti-PrP antibody 6H4 raised against bovine recombinant PrP, recognizing amino acids 144–152 of human PrP (1∶1000 in blocking buffer, Prionics, Schlieren, Switzerland). After incubation for 1 hour at room temperature with an HRP-conjugated anti-mouse secondary antibody (1∶5000 in blocking buffer), signal was detected using ECL femto reagent (Thermo Scientific, Rockford, USA) and visualized with a BioRad ChemiDoc imaging station and glycotpyes were evaluated using published protocols [41].

### Sodium phosphotungstic acid (NaPTA) precipitation

NaPTA precipitation was done according to published protocols [21], [42]) with minor changes. Muscle tissue (100 mg) was dissociated in 900 µl buffer (25 mM HEPES (pH 7.2), 0.3 M sucrose and 53,6 µg Liberase Blendzyme 2 (Roche, Mannheim, Germany)). Samples were incubated for 30 min at 37°C and homogenised (FastPrep FP120, Qbiogene, Cedex, France). 500 µl of 10% (w/v) tissue homogenates and 500 µl of 4% (w/v) sarkosyl-PBS were vortexed and incubated for 10 min at 37°C with constant agitation. Benzonase (Novagen, Merck, Nottingham, UK (50 U/ml)) and 1 mM MgCl_2_ were added and incubated at 37°C for 30 min with vigorous shaking. 81.3 µl of a pre-warmed (to 37°C) 4% (w/v) NaPTA/170 mM MgCl_2_ solution (pH 7.4) was added, vortexed and incubated with vigorous agitation for 30 min at 37°C. Samples were centrifuged at 25,000 g for 30 min; pellets were resuspended in 22.5 µl of 0.1% sarkosyl-PBS, followed by proteinase K digestion (Roche, Mannheim, Germany) with a final concentration of 20 µg/ml for 1 hour at 37°C and processed for western blotting.

### ELISA

ELISA measurements were performed using a modified version of the Bio-Rad test (Platelia®BSE, TeSeE®) that is also used for the *post mortem* diagnosis of BSE in cattle according to published protocols [34]. This kit uses two different monoclonal antibodies allowing a sensitive detection of denatured PrP. PrP^Sc^ was purified and concentrated from 50 mg fresh wet tissue homogenate using the TeSeE® purification kit following the manufacturer's instructions. This procedure included a PK treatment (0.4 µg/mg of tissue) and a centrifugation step to concentrate PrP^Sc^. The corresponding pellet was denatured at 100°C in the presence of a mixture of a chaotropic agent and a detergent. After fivefold dilution in an appropriate buffer, denatured PrP^Sc^ was successively reacted with capture antibody (Sha-31) and tracer antibody (Pri-308) that were specific for the detection of primate prion protein.

### Paraffin-embedded tissue (PET)-Blot

PET blots were performed according to published protocol [43]. Briefly, formalin-fixed and decontaminated tissue was embedded in paraffin, sectioned (5 µm), collected on a prewetted nitrocellulose membrane (Bio-Rad, Hercules, USA) and completely dried at 55°C. After deparaffinization and rehydration, the membrane was washed in Tween 20 (0.1%) and dried for PrP detection.

Prewetted membranes were digested with proteinase K (250 µg/ml) for 8 hours at 55°C. After washing, proteins were denatured with 4 mol/l guanidine isothiocyanate for 15 minutes, washed again and immunodetection was performed after preincubation in blocking solution. Monoclonal antibody 12F10 recognizing amino acid 142–160 of human PrP (kindly provided by J. Grassi; [44], commercially available through SPIbio, Montigny, France) was used as primary antibody for at least 1 hour. Samples were washed again, incubated with alkaline-phosphatase-coupled rabbit anti-mouse antibody (Dako, Glostrup, Denmark) and detected using NBT/BCIP. Blots were evaluated with an Olympus dissecting microscope (Olympus, Hamburg, Germany).

### Morphological analyses

Brain (cerebellum) or muscle tissue (tongue) was fixed with 4% buffered formalin, inactivated by 98% formic acid for one hour and embedded in paraffin. Sections were cut (3 µm) deparaffinised and stained with haematoxylin and eosin according to standard protocols [21].
